# Predictors of early breastfeeding initiation among mothers of children under 24 months of age in rural part of West Ethiopia

**DOI:** 10.1186/s12889-015-2420-z

**Published:** 2015-10-21

**Authors:** Tsedeke Wolde Hailemariam, Emiru Adeba, Alem Sufa

**Affiliations:** Nutrition Unit, Population & Family Health Department, College of Public Health and Medical Sciences, Jimma University, P.O. Box 378, Jimma, Ethiopia; Department of Public Health, College of Medical and Health Sciences, Wollega University, P.O. Box 395, Nekemte, Ethiopia; Reproductive Health Unit, Department of Public Health, College of Medical and Health Sciences, Wollega University, P.O. Box 395, Nekemte, Ethiopia

**Keywords:** Breastfeeding, Breastfeeding initiation, Predictors, West Ethiopia

## Abstract

**Background:**

The World Health Organization recommends initiation of breastfeeding within the first hour after childbirth. In developing countries alone, early initiation of breastfeeding could save as many as 1.45 million lives each year by reducing deaths mainly due to diarrheal disorders and lower respiratory tract infections in children. The current study aimed to determine the rate and the predictors of breastfeeding initiation in East Wollega Zones of West Ethiopia.

**Methods:**

A community-based, cross-sectional study was conducted from April to May 2014 among 594 mothers who had children less than 24 months. Multi stage cluster sampling method was used to select the study population. Eligible mothers were invited to interview using pretested questionnaires to gather data regarding sociodemographics, health-related variables, breastfeeding initiation, and current breastfeeding practices. A multivariable logistic regression analysis was used to identify independent predictors of early initiation of breastfeeding after controlling for confounding variables.

**Results:**

A sample of 593 mothers was included in the study. Breastfeeding was initiated by 83.1 % of mothers within the first hour of childbirth. Being a housewife (AOR (95 % CI) = 2.48 (1.54- 3.99)) and infant received colostrum (AOR (95 % CI) =2.22 (1.08-4.55)) were significant positive predictors for early breastfeeding initiation as revealed by logistic regression. The multivariable logistic regression analysis showed that the mothers who had no radio and/or TV in the household (AOR (95 % CI = 0.55 (0.35-0.88)), were not exposure to health information (AOR (95 % CI) = 0.44 (0.25-0.75)), and infants were provided with prelacteal feeds (AOR (95 % CI)=0.30 (0.14-0.65)) were less likely to initiate breastfeeding.

**Conclusions:**

The rate of timely initiation of breastfeeding was high. Breastfeeding promotion program is essential to encourage the practice of timely initiation of breastfeeding, and reduce the practice of providing prelacteal feeds within three days of life. Thus appropriate health information is vital to boost early initiation of breastfeeding.

## Background

The World Health Organization (WHO) and UNICEF recommend initiation of breastfeeding within the first hour after birth [1].

Early initiation of breastfeeding, particularly within the first hour, is important for both the mother and the child, as suckling stimulates the release of hormones which help produce milk. It also stimulates the contraction of the uterus after childbirth, thus decreasing the likelihood of postpartum hemorrhage. The milk produced from the breast in the first three days after delivery, known as “colostrum,” is rich in natural antibodies that will help protect the newborn against infections [2].

In developing countries alone, early initiation of breastfeeding could save as many as 1.45 million lives each year by reducing deaths mainly due to diarrheal disorders and lower respiratory tract infections in children [3].

The 2011 Ethiopia Demographic and Health Survey (EDHS) reported that nationally, 52 % of mothers initiated breastfeeding within one hour of childbirth [4]. A recent study from Nekemte town of East Wollega Zone [5] reported a higher rate (88.5 %) of early initiation breastfeeding.

Giving prelacteal has been shown to delay the initiation of breastfeeding [6]. By interfering with breastfeeding during this period, prelacteal feeding diminishes the immunological benefits a newborn receives, thus increasing his/her susceptibility to infection. In addition, prelacteal feeding can be a direct cause of illness by exposing infants to contaminated feeds, utensils, water, or hands. Prelacteal feeding may also affect neonatal health by disrupting the priming of the gastrointestinal tract [7]. Predictors of early initiation of breastfeeding have not been previously reported in the study area. To address this gap, this study aimed to estimate the magnitude of timely initiation of breastfeeding and to identify potential factors associated with initiation of breastfeeding among West Ethiopia mothers. The current study could help to policy makers and implementers on breastfeeding promotion programs to design appropriate strategies to tackle the problem.

## Methods

### Study design, setting and participants

A Community based cross-sectional study design was conducted in three districts (Sibu Sire, Arjo Gudetu & Arjo Jimma) of East Wollega zone, West Ethiopia from April 01 to May 01, 2014. Capital town of the zone, which is located at about 331kms West of Addis Ababa. East Wollega zone is one of the 17 zones of Oromia regional state with total population of 1,230,402; 615,641 are females. Eighty six percent (n = 1,061,120) [8] of the population live in the rural areas. All rural mothers who have children less than 24 months were considered as source population. Since studies showed that rural women are less likely to initiate breastfeeding than women in urban areas. Therefore, this study aimed to focus on identifying the determinant factors of early breastfeeding initiation among rural women. Mothers who were critically ill during the data collection period were excluded from the study.

To determine the sample size, a single population proportion formula with a proportion of mothers who started breastfeeding within one hour of birth in Arbaminch (South Ethiopia region) was 57 % [9], a confidence level of 95 %, and a 5 % degree of precision, were used. A design effect of 1.5 and non-response rate of 5 %, were also used. The final sample size calculated was 594 mothers who have children less than 24 months.

Multi stage cluster sampling method was used to select and approach the study population. In the first stage of the sampling, the three districts randomly selected from twenty one districts of East Wollega zone. In stage two, nine clusters/kebeles (the smallest administrative unit in Ethiopia) were selected by assigning probability proportional to the population size from thirty rural kebeles. In each district, the total populations were calculated and the names of the kebeles and population size of the districts. Ethiopia Demographic and Health Survey (EDHS) 2011 data [4] were used for identification of the names of the kebeles and population size of each district. In each kebele census was conducted to identify households with children aged less than 24 months. Once household with children aged less than 24 months were identified. In each kebele a number of households with probability proportional to the population size were allocated. Then systematic random sampling technique was used to reach individual households. The first houses were identified from the central places which were kebele administrative offices. By rotating a pen on a ground, where the sharp end indicating was taken to be the first household. Data collectors counted the total number of houses between the kebele administrative offices and the boundary of the kebeles. The total number of the children in the study population divided by the number of clusters/kebeles (9) determines the sampling interval. The subsequent households were selected by using the sampling intervals. Data collectors were continuing their interview until the calculated samples were obtained. If the household had more than one child in this age group, one child was randomly selected for participation in the study.

### Data collection

The data were collected by using a structured and pre-tested interview questionnaire. The questionnaire was initially prepared in English and then has been translated into the local language, Afan Oromo, by fluent speakers of both languages and again it was translated back into English to check its consistency. Six clinical nurses who speak local languages were employed in the data collection process. Three BSc nurses were selected as a supervisor. Training was given to the data collectors and supervisors for three consecutive days on the objectives of the study, the contents of the questionnaire, particularly on issues related to the confidentiality of the responses and the rights of respondents. One week prior to the data collection, a pre-test was conducted at Diga district in 30 (5 %) of the sample size. Additional modifications were made to the questionnaire in terms of terminologies and formatting based on the pretest findings. Every day, all collected data were checked for their completeness, clarity and consistency by supervisor and principal investigator. Any unclear and ambiguous data were corrected at that time by the supervisor and principal investigator.

### Definition of variables

#### Outcome variable

In this study, mothers were asked about whether they had initiated breastfeeding and how long after giving birth. The outcome variable (timely initiation of breastfeeding) was expressed as the proportion of infants who first suckled within 1 hour of birth. The main outcome variable (initiated breastfeeding with one hour of birth = 1 and did not initiate breastfeeding with one hour of birth = 0).

#### Independent variables

The study used one major questionnaire with four sections, namely socio-demographic factors, maternal factors, child related factors and characteristics of child feeding practices were included in the survey based on the literature review and adapted from 2011 Ethiopia DHS (4).

### Data analysis

The data were entered in double, cleaned and checked for missing values and outliers, and analyzed using IBM SPSS statistics for windows, version 20.0 [10]. Simple descriptive analyses were used to describe the initiation of breastfeeding practices and characteristics of the study population. Multivariate analysis of logistic regression was performed to analyze the factors associated with initiation of breastfeeding outcomes, while controlling for the other covariates. The final models present the results as odds ratios (OR) with 95 % confidence intervals (95 % CI). All of the results were considered statistically significant at P <0.05.

### Ethical considerations

Ethical clearance was obtained from Wollega University Institutional Review Board for Ethical approval and permission. At the time of data collection, a verbal and written consent was taken from the participants to confirm whether they are willing to participate or to obtain oral consent. Information was collected after securing consent from study participants. Data obtained from each study participant were kept confidential.

## Results

### Socio-demographic characteristics of the participants

Out of 594 participants intended to be included, a complete response was obtained from 593 (99.8 %) respondents. The majority (37.8 %) of mothers were in the age group 20–24 years. The mean age of mothers was 25.7 years. The highest proportion of mothers (36.9 %) had attended primary school (1–8 grades). Slightly more than half (52.1 %) of the mothers were housewives. Less than a half (41.1 %) of the study participants had medium wealth tertiles (Table 1).

**Table 1:** Socio-demographic characteristic of participants in rural areas of West Ethiopia, April to May, 2014 (*n* = 593)

Variables (*n* = 593)		Frequency (%)
Age of child in months	1-5	152 (25.6)
	6-11	181 (30.5
Sex of child	12-23	260 (43.9)
	Male	299 (50.4)
Age of mother in years	Female	294 (49.6)
	15-19	55 (9.3)
	20-24	224 (37.8)
	25-29	174 (29.3)
	30-34	91 (15.3)
Family size	35+	49 (8.3)
	1-5	464 (78.2)
	>5	129 (21.8)
Paternal Educational status	Mean family size	5
	Illiterate	97 (16.4)
	Primary complete	205 (34.6)
	Secondary complete	136 (22.9)
Maternal Educational status	Above secondary	155 (26.1)
	Illiterate	160 (27)
	Primary complete	219 (36.9)
	Secondary complete	113 (19.1)
Occupation (mothers)	Above secondary	101 (17)
	House wife	309 (52.1)
	Government employee	75 (12.6)
	Farmer	18 (13.7)
	Merchant	110 (18.5)
	Others^a^	18 (3)
Variables (n = 593)		Frequency (%)
Ethnicity	Oromo	540 (91.1)
	Amahara	41 (6.9)
	Guraghe	10 (1.7)
Religion	Others^b^	2 (0.3)
	Orthodox	236 (39.8)
	Protestant	324 (54.6)
	Muslim	27 (4.6)
Wealth index of household	Others^c^	6 (1)
Poor		182 (30.7)
Medium		244 (41.1)
High		167 (28.2)
Owner radio/TV		
Yes		330 (55.6)
No		263 (44.4)

^a^ = student, daily laborer, ^b^ = Shinasha, Wolaita ^c^ = catholic, Adventist

### Prevalence of breastfeeding initiation related practices

The study participants were witnessed that majority of the children 96 % were ever breastfed at some point in the past. From those who were ever breastfed, 83.1 % of the mothers initiated breastfeeding within one hour of birth. Nearly all of the mothers in this study reported feeding colostrum to their infants (91.2 %). A significant proportion (83.6 %) of the mothers was received advice on breastfeeding after delivery from health worker. A total of 40 (6.7 %) of mothers had reported of providing prelacteal feeds to their newborn infants. The proportion (33.2 %) of mothers who were practiced bottle feeding for their infants (Table 2).

**Table 2:** Practice of timely initiation of breastfeeding in rural areas of West Ethiopia, April to May, 2014 (*n* = 593)

Variables (593)	Number (%)
Ever breast-fed	
Yes	569 (96.0)
No	24 (4.0)
Initiation of breastfeeding within one hour	
Yes	473 (83.1)
No	96 (16.9)
Frequency of breast-feeding per 24 hours	
<8 times	45 (8)
≥8 times	524 (92)
Giving colostrum	
Yes	541 (91.2)
No	52 (8.8)
Advice on BF received after delivery	
Yes	496 (83.6)
No	97 (16.4)
Prelacteal feeding	
Yes	40 (6.7)
No	553 (93.3)
Bottle feeding	
Yes	197 (33.2)
No	396 (66.8)

### Predictors of early breastfeeding initiation

Table 3 shows the results of the multiple logistic regression analysis based on the significant variables obtained from the bivariate analyses. Being a housewife, absence of radio and/or TV, colostrum feeding, pre-lacteal feeding practice and advice not given for the mother after birth were associated with early initiation of breastfeeding.

**Table 3:** Multivariable logistic regression analysis showing factors associated with early initiation of breastfeeding among mothers in rural areas of West Ethiopia, April to May, 2014 (*n* = 593)

	Initiation of BF within one hour		95 % CI	
Variables (*n* = 593)	Yes	No	COR	AOR
	*N* (%)	*N* (%)		
Maternal Occupation				
Housewife	266 (89.3)	32 (10.7)	2.57 (1.62-4.08)^c^	2.48 (1.54- 3.99)^c^
Business women^a^	207 (76.4)	64 (23.6)	1.00	1.00
Owner Radio/TV				
Yes	277 (87.1)	41 (12.9)	1.00	1.00
No	196 (78.1)	55 (21.9)	0.53 (0.34-0.82)^c^	0.55 (0.37-0.88)^c^
Colostrum feeding				
Yes	443 (84.5)	81 (15.5)	2.74 (1.41-5.31)^c^	2.22 (1.08-4.55)^c^
No	30 (66.7)	15 (33.3)	1.00	1.00
Advise after delivery on BF				
Yes	411 (85.8)	68 (14.2)	1.00	1.00
No	62 (68.9)	28 (31.1)	0.37 (0.22-0.61)^c^	0.44 (0.25-0.75)^c^
Prelacteal feeding				
Yes	21 (60.0)	14 (40)	0.27 (0.13-0.56)^c^	0.30 (0.14-0.65)^c^
No	452 (84.6)	82 (15.4)	1.00	1.00

^a^Business women = farmer, merchant, government employee

^b^Statistically significant CI., *CI* confidence interval, *COR* crude odds ratio, *AOR* adjusted odds ratio

Independent variables entered in the initial model: sex of child, maternal age, mother’s occupation, owner radio/TV, maternal education, wealth index, colostrum feeding, advise, and prelacteal feeding. [d.f.13 Wald chi square value:35.034 (*p* < 0.001); Hosmer-Lemeshow goodness of fit test: 0.456]. Model 1: mother’s occupation, owner radio/TV, colostrum feeding, advice, and prelacteal feeding. [d.f. 5; Wald chi square value; 32.315 (*p* < 0.000); Hosmer-Lemeshow goodness of fit test: 0.611]. Maternal characteristics examined as control variables included age and education. We also adjusted for sex of child in the analyses

Being a housewife was found to be 2.5 times more likely to initiate breastfeeding within one hour of delivery compared to their counterparts (AOR (95 % CI) = 2.48 (1.54- 3.99). Colostrum feeding practice was another important predictor of early initiation of breastfeeding. Mothers who fed colostrum after birth were 2.2 times more likely to initiate breastfeeding within one hour of birth (AOR (95 % CI) = 2.22 (1.08-4.55)).

Those participants who had no radio and/or TV were 45 % times less likely to initiate breastfeeding with the recommended time compared to their counterparts (AOR (95 % CI)=0.55 (0.37-0.88)). Mothers who did not receive health information on breastfeeding after delivery from health personnel were 56 % times less likely to initiate early breastfeeding (AOR (95 % CI) = 0.44 (0.25-0.75)).

Likewise women who gave pre-lacteal feeding were 70 % times less likely to initiate early breastfeeding within one hour of childbirth (AOR (95 % CI) = 0.30 (0.14-0.65)) (Table 3).

## Discussion

The study aimed to determine the prevalence and predictor factors with timely initiation breastfeeding. Timely initiation of breastfeeding is important for both the mother and the child [4]. This study reported a prevalence of breastfeeding initiation (83.1 %) which is lower than Nekemte town study (88.5 %) [5]. The finding was similar to finding from Karen refugees [11]. But, it is much higher than other similar studies done national report of Ethiopia (52 %), North Jordan (86.6 %), Saudi Arabia, Arbaminch area (South Ethiopia), Nepal, West Nepal (72.2 %), Nigeria (45 %), and Ghana (41 %) [4, 12–18]. The possible reason for high rate of initiation of breastfeeding in this population might be due to the variation in culture. Early initiation of breastfeeding is important for both the mother and the child. Early suckling stimulates the release of prolactin, which helps in the production of milk and oxytocin, which is responsible for the ejection of milk and stimulates the contraction of the uterus after childbirth [7]. A review of the literature between 1985 and 2009 [19] concludes that educational programmes in the context of on-going ‘personal contact’ with a health professional are effective in promoting the initiation and prevalence of breastfeeding. Thus, comprehensive information should be given for all women in the reproductive age group on importance of early initiation of breastfeeding.

Global strategy on infant and young child feeding, recommends feeding colostrums. Colostrum is produced in the first few days after delivery and provides natural immunity to the infant. It is recommended that children be fed colostrums immediately after birth and continue to be exclusively breastfed even if the regular breast milk has not yet let down [4]. Moreover, there is a good practice in this study in which 91.2 % of mothers gave colostrums to their baby. This finding was broadly in line with the finding in Nepal (86 %) [16]. However, it was higher than a report on Nigerian women, which was 24 % [20]. The finding was also similar with previous study conducted in Ethiopia [14]. Health extension workers deployed in rural areas might be responsible for wide practice of colostrum feeding in the study area. Early initiation of breastfeeding prevents neonatal and infant deaths largely by reducing the risk of infectious diseases. This risk is reduced because: Colostrum, the first breast-milk contains a large number of protective factors that provide passive and active protection to a wide variety of known pathogens. Colostrum is particularly rich in these protective factors and its ingestion within the first hour of life prevents neonatal mortality [21].

In the current study, housewives were 2.5 times more likely initiate breastfeeding within one hour after birth than women who had business outside home. It might be women working outside home were not interested to start breastfeeding because of possibility of discontinuation of breastfeeding due to work.

Similar with this study previous studies showed that advice received/knowledge on early initiation of breastfeeding and having radio/TV were associated with initiation of breastfeeding within one hour of birth [8, 14]. Obviously exposure to different source of information plays a great role for women to practice optimal breastfeeding including early initiation of breastfeeding. Thus, information provision during pre-natal and post-natal care on early initiation of breastfeeding should be strengthened.

In this study, mothers who giving prelacteal feeds to infants (e.g. butter, water, glucose water, teas and herbal preparations) within three days of life was 70 % times less likely to initiate breastfeeding within one hour after birth. The finding was consistent with findings from Saudi Arabia [13]. Prelacteal feeding result in the baby receiving insufficient breast milk possibly leading to lactation failure and weakens the suckling stimulus. The finding highlights the importance of discouraging prelacteal feeding for the first three days of life.

Major strengths of this study were the community based approach and random selection of the study households. This may made generalization possible to the study communities as an attempt was made to identify randomized households and women with their children from the study communities. However, the cross-sectional nature of this study limited drawing causal inferences from the association between the factors and timely initiation of breastfeeding practices. There might be retrospective recall bias on breastfeeding practices as the mothers could forget when they introduced complementary foods.

## Conclusions

This study revealed that the proportion of timely initiation breastfeeding was comparatively high. A significant number of mothers had positive attitude towards timely initiation breastfeeding. Providing colostrums within the first hour could have an influence on timely initiation breastfeeding. Being a housewife, infant received colostrum, infant received pre-lacteal feeds, absence of radio and/or TV in the household, non exposure to health information on breastfeeding after delivery were found to be significantly associated with early breastfeeding initiation within an hour. Breastfeeding promotion program is essential to encourage the practice of timely initiation of breastfeeding and reduce the practice of providing prelacteal feeds. Therefore, appropriate health information is vital to boost early initiation of breastfeeding.

## References

[1] World Health Organization, UNICEF (2003). Global Strategy for Infant and Young Children Feeding.

[2] UNIFPA (2010). Egypt Demographic and Health Survey 2008 Breastfeeding Status in Egypt.

[3] Lauer JA, Betran AP, Barros AJ, De Onis M (2006). Deaths and years of life lost due to suboptimal breast-feeding among children in the developing world: a global ecological risk assessment. Public Health Nutr.

[4] Central Statistical Agency [Ethiopia] and ICF International (2012). Ethiopia Demographic and Health Survey 2011.

[5] Tsedeke W, Tadesse B, Eyasu E (2014). Prevalence and Determinants of Timely Initiation of Breastfeeding among Lactating Mothers of Urban Dwellers in Western Ethiopia. Int J Food Sci Qual Manag.

[6] Ogah AO, Ajayi AM, Akib S, Okolo SN (2012). A cross-sectional study of pre-lacteal feeding practice among women attending Kampala International University Teaching Hospital Maternal and Child Health Clinic, Bushenji,Western Uganda. Asian J Med Sci.

[7] Goldman AS (2000). Modulation of the gastrointestinal tract of infants by human milk. Interfaces and interactions. An evolutionary perspective. J Nutr.

[8] FDREC (2008). Summary and statistical report of the 2007 population and Housing census.

[9] Tamiru D, Bogale B, Merdikios B (2013). Feeding patterns and factors associated with exposure to suboptimal breast-feeding practices in rural communities of Arba Minch Zuria. Ethiopia, global health perspective.

[10] Corporation IBM (2011). IBM SPSS statistics for Windows version 20.0.

[11] White AL, Carrara VI, Paw MK, Malika, Dahbu CP, Gross MM, et al. High initiation and long duration of breastfeeding despite absence of early skin-to-skin contact in Karen refugees on the Thai-Myanmar border: a mixed methods study. Int Breastfeed J. 2012;7:19. 10.1186/1746-4358-7-19 PMC3547777 23241099

[12] Örün E, Songül SY, Madendaù Y, Üstünyurt-Eras Z, Kutluk U, Yurdakök K (2010). Factors associated with breastfeeding initiation time in a Baby-Friendly Hospital. Turk J Pediatr.

[13] El-Gilany A-H, Sarraf B, Al-Wehady A (2012). Factors associated with timely initiation of breastfeeding in Al-Hassa province, Saudi Arabia, Eastern. Mediterr Health J.

[14] Tamiru D, Mohammed S (2013). Maternal knowledge of optimal breastfeeding practices and associated factors in rural communities of Arba Minch Zuria. Int J Nutr Food Sci.

[15] Adhikari M, Khanal V, Karkee R, Gavidia T (2014). Factors associated with early initiation of breastfeeding among Nepalese mothers: further analysis of Nepal Demographic and Health Survey, 2011. Int Breastfeed J.

[16] Chandrasekhar TS, Joshi HS, Binu VS (2007). Breast-feeding initiation and determinants of exclusive breast-feeding: A questionnaire survey in an urban population of western Nepal. Public Health Nutr.

[17] Agunbiade OM, Ogunleye OV (2012). Constraints to exclusive breastfeeding practice among breastfeeding mothers in Southwest Nigeria: implications for scaling up. Int Breastfeed J.

[18] LINKAGES, Experience: Result final report http://www.linkagesproject.org/media/publications/Results11–06.pdf] web site Ghana, 2006.

[19] Ibanez G, DeReynal De Saint Michel C, Denantes M, Saurel Cubizolles MJ, Ringa V, Magnier AM (2012). Systematic review and meta-analysis of randomized controlled trails evaluating primary care-based interventions to promote breastfeeding in low-income women. Fam Pract.

[20] Davies A, Anita A (1997). Socio cultural factors and the promotion of exclusive breastfeeding in rural Yoruba communities of Osun State, Nigeria. J Soc Sci Med.

[21] WHO (2010). Early Initiation of Breastfeeding: the Key to Survival and Beyond.

